# Effectiveness of Gagné’s 9 Events of Instruction in health professions education: a systematic review and meta-analysis

**DOI:** 10.3389/fmed.2025.1522830

**Published:** 2025-04-08

**Authors:** Yue Li, Zhengjv Liang, Zhongyan Li, Yuhuan Yu, Qing Yang, Xiao Li

**Affiliations:** ^1^Nursing College of Yunnan University of Traditional Chinese Medicine, Kunming, Yunnan, China; ^2^Honghe Prefecture Third People’s Hospital, Gejiu, Yunnan, China; ^3^DeHong Vocational College, DeHong, Yunnan, China; ^4^Honghe Prefecture Yunnan Central Hospital, Gejiu, Yunnan, China

**Keywords:** Gagné’s 9 Events of Instruction, Gagné’s instructional theory, clinical medicine, health professions education, medical students, nursing education, traditional teaching, meta-analysis

## Abstract

**Objective:**

This review assesses the effectiveness of Gagné’s 9 Events of Instruction in improving theoretical scores and clinical practice abilities in medical education.

**Methods:**

This study followed the Preferred Reporting Items for Systematic Reviews and Meta-Analyses (PRISMA) guidelines. Two researchers conducted a comprehensive search of Chinese and English electronic databases (Web of Science, PubMed, Embase, Cochrane Library, CNKI, VIP, Wanfang). The participants included clinical medical students, nursing students, specialized medical students, and medical interns, among others related to healthcare. Educators used Gagné’s 9 Events of Instruction to guide these populations in theoretical learning and/or daily clinical practice. The search was conducted from the inception of the databases to February 27, 2025. Two researchers independently identified, selected, and extracted data from the studies, assessed the quality using the Cochrane Risk of Bias tool, and performed meta-analyses using RevMan 5.4 and Stata 17.0.

**Results:**

A total of 11 studies involving 825 participants were included in the meta-analysis, including 5 RCTs and 6 CSs. In the cumulative meta-analysis, compared with the traditional LBL model, Gagné’s 9 Events of Instruction significantly improved learners’ KES (SMD 1.55, 95% CI: 0.81 to 2.29; *p* < 0.00001), PS (SMD 1.83, 95% CI: 1.19 to 2.47; *p* < 0.00001), LC (OR 4.92, 95% CI: 3.13 to 7.73; *p* < 0.0001), and TS (OR 7.86, 95% CI: 3.22 to 19.20; *p* < 0.0001).

**Conclusion:**

The meta-analysis indicated that, compared to traditional medical teaching models, Gagné’s 9 Events of Instruction are significantly effective in health professions education and can effectively enhance learners’ KES, PS, LC, and TS.

## 1 Introduction

Robert Mills Gagné proposed that learning is a dynamic process formed by interacting internal cognitive activities and the external environment, through which learners construct a comprehensive cognitive structure containing meaning, attitude, and motivation (1). Gagné creatively put forward the “9 Events of Instruction” model to optimize this process, closely integrating instructional design with human cognitive processing mechanisms and emphasizing the correspondence between external teaching interventions and internal learning stages (2).

Gagné’s 9 Events of Instruction construct a complete teaching loop in a linear sequence, with the core of activating and guiding internal cognitive processing through external events (3). Specifically, the model starts with “Gain Attention” to stimulate learning motivation, then establishes cognitive expectations through “Inform Learners of Objectives”; subsequently, it achieves knowledge connection with “Stimulate Recall of Prior Learning,” inputs new information with “Present Stimulus,” and promotes meaning construction with “Provide Learner Guidance.” In the output phase, “Elicit Performance” tests the level of understanding, “Provide Feedback” reinforces correct responses, “Assess Performance” measures the degree of goal achievement, and finally, “Enhance Retention and Transfer” realizes the internalization and application of knowledge (4). This theoretical framework reveals the scientific logic of teaching activities. It provides a systematic and operable path for modern instructional design, with its influence continuously permeating fields such as educational technology and curriculum development.

The application research of Gagné’s 9 Events of Instruction is mainly focused on university subject studies (5–7). It has broken the traditional teaching design where educators unilaterally impart knowledge and learners passively accept it. Educators, based on different subjects and with learners as the main body, advocate cultivating learners’ interest in learning and giving full play to their initiative during the teaching process. The learning design focuses on breaking down complex knowledge into simple knowledge, making abstract knowledge more tangible, and presenting it in the best way that learners can accept, following a process from easy to difficult, shallow to deep, and step by step (8). In the final stage, assessment and feedback are carried out to point out the existing shortcomings and problems, further deepening learning (9).

Health professions education requires high theoretical learning ability and practical operation skills. At the same time, the content of health professions education courses is more abstract and complex than other subjects, making learning more challenging (10). The traditional teaching method (Lecture-based Learning) is mainly educator-centered, with learners passively listening under the conventional teaching guidance, leading to unstable knowledge mastery and poor overall teaching effect (11, 12). Moreover, the traditional teaching method focuses on textbook content, emphasizing theoretical knowledge while neglecting practical training. This method is increasingly unsuitable for medical teaching emphasizing clinical practice (13).

How to change teaching methods to make complex medical knowledge easy to understand and leave a deep impression on learners with fragmented knowledge is a problem faced by current teaching reform (14). Traditional teaching models can no longer fully adapt to the continuous development of medical education and the rapidly increasing volume of course knowledge. Gagné’s 9 Events of Instruction has achieved good educational results in health professions education (15, 16), but its application is relatively late and lacks high-level evidence. This study aims to evaluate the effectiveness of Gagné’s 9 Events of Instruction compared to traditional medical teaching in health professions education, including KES, PS, LC, and TS.

## 2 Methods

This study followed the Preferred Reporting Items for Systematic Reviews and Meta-Analyses (PRISMA) guidelines (17) and included a systematic review and a meta-analysis. The completed PRISMA checklist is provided as Supplementary material S1. Additionally, this review was conducted by the Cochrane Handbook for Systematic Reviews of Interventions (18).

### 2.1 Systematic review methodology

#### 2.1.1 Data sources and search strategy

Computer searches were conducted in China National Knowledge Infrastructure (CNKI), Chinese Science and Technology Journal Database (VIP), Wanfang Database (WANGFANG DATA), as well as PubMed, Web of Science, Embase, and Cochrane Library. The search period was from the inception of the databases to February 27, 2025. For the Chinese search, taking CNKI as an example: “(Subject: Gagné’s instructional theory) OR (Subject: Gagné’s theory) OR (Subject: Nine-event teaching method) OR (Subject: Gagné’s teaching model)” AND “(Title, Keywords, Abstract: Medicine (fuzzy)) OR (Title, Keywords, Abstract: Clinical medicine (fuzzy)) OR (Title, Keywords, Abstract: Nursing (fuzzy)) OR (Title, Keywords, Abstract: Specialized medical students (fuzzy)) OR (Title, Keywords, Abstract: Interns (fuzzy)).” For the English search, taking PubMed as an example: (((((Gagne’s 9 Events of Instruction [Title/Abstract]) OR (Gagne’s Nine Steps of Instructional Design [Title/Abstract])) OR (Gagne’s Model of Instructional Design [Title/Abstract])) OR (Gagne’s Model of Instructional Design [Title/Abstract])) OR (nine events [Title/Abstract])) AND (((((Education) OR (Nursing)) OR (clinical)) OR (medicine)) OR (Student)). Additionally, we conducted partial searches on Google Scholar to review relevant gray literature. We also used the “snowballing method” to screen the reference lists of relevant studies to identify further studies that met the criteria. The search strategy is provided as Supplementary material S2.

#### 2.1.2 Inclusion and exclusion criteria

Two researchers independently screened the retrieved literature. When the two researchers disagreed, they first discussed to reach a decision. A third researcher made the judgment if consensus still could not be reached. The following inclusion criteria were used according to PICOS (Table 1).

**Table 1:** Inclusion criteria based on PICOS in this systematic review and meta-analysis.

Population	Clinical medical students, nursing students, specialized medical students, and medical interns, among others related to healthcare (34, 42).
Intervention	Gagne’s 9 Events of Instruction: (Gain attention, Inform learners of objectives, Stimulate recall of prior learning, Present stimulus, Provide learner guidance, Elicit performance, Provide feedback, Assess performance, Enhance Retention and Transfer)
Comparison	Lecture-based Learning (LBL): It is defined as a teaching model that is educator—centered and primarily focused on one—way knowledge transmission.
Outcomes	Primary Outcomes: Knowledge Examination Score (KES): Quantitatively assessed through standardized written tests or course—final exams. Practice Score (PS): Scores based on simulated operations, clinical skills assessments, or Objective Structured Clinical Examinations (OSCE).
	Secondary Outcomes: Learning Compliance (LC): Measured by attendance rate, classroom participation, or task completion rate. Teaching Satisfaction (TS): Assessed using an efficient self-administered satisfaction questionnaire.
Study design	Only Randomized Controlled Trials (RCTs) and prospective Cohort Studies (CS) were included to control for confounding factors and ensure the rigor of causal inference.

Exclusion Criteria: Review articles or case reports; Lack of primary outcomes or insufficient data; Non-comparative studies; Non-medical courses; Non-English and non-Chinese articles.

#### 2.1.3 Risk of bias assessment

According to the Cochrane bias risk assessment recommendations, the revised tool RoB 2.0 for assessing the risk of bias in RCTs (19) was used to evaluate the bias risk in five domains (randomization process, deviation from intended interventions, missing outcome data, measurement of the outcome, and selection of the reported result) of the studies, and finally classified the bias risk of the studies into low, medium, or high risk. For non-RCTs, the risk of bias in non-randomized studies tool ROBINS-I was used to evaluate the bias risk of the primary outcomes (20), which was assessed from seven domains including confounding bias, selection bias of participants, classification bias of interventions, deviation bias from intended interventions, bias due to missing data, bias in measurement of outcomes, and selective reporting bias, and finally classified the bias risk of the studies into low, medium, high, or serious risk. Two independent researchers completed the double-blind assessment, and when consensus could not be reached, a third senior researcher made the final arbitration.

### 2.2 Meta-analysis method

#### 2.2.1 Data extraction

Data extraction was preliminarily conducted using an Excel worksheet, including the first author’s surname, year, study design, sample size of the included studies, education level, majors, course name, course type, and outcome measures.

#### 2.2.2 Statistical analysis

Meta-analyses were conducted using RevMan 5.4 and Stata 17.0 software. Since different studies may use different rating systems for continuous variables such as KES and PS, we have chosen the Standardized Mean Difference (SMD) and 95% Confidence Interval (CI) as the measure of effect. The SMD eliminates the impact of different rating scales by dividing the mean difference by the pooled standard deviation. For categorical variables such as LC and TS, we used the Odds Ratio (OR) and 95% CI for the combined analysis. A *p*-value less than 0.05 was considered statistically significant (21). We conducted heterogeneity tests on the included studies. If there was no significant heterogeneity (I^2^ < 50%, *p* ≥ 0.05), we used the fixed-effects model for the meta-analysis; if there was significant heterogeneity (I^2^ ≥ 50%, *p* < 0.05), we used the random-effects model (22).

#### 2.2.3 Test for publication bias

A funnel plot was used to detect publication bias in the study results. A symmetrical distribution of the indicators in the funnel plot suggests the absence of publication bias.

#### 2.2.4 Sensitivity analysis

We used the leave-one-out approach to conduct sensitivity analyses for the primary outcomes. The direction and magnitude of the pooled estimate did not change significantly when any particular study was removed, indicating that the meta-analysis’s results are relatively stable.

## 3 Results

### 3.1 Search results

**Figure 1:**
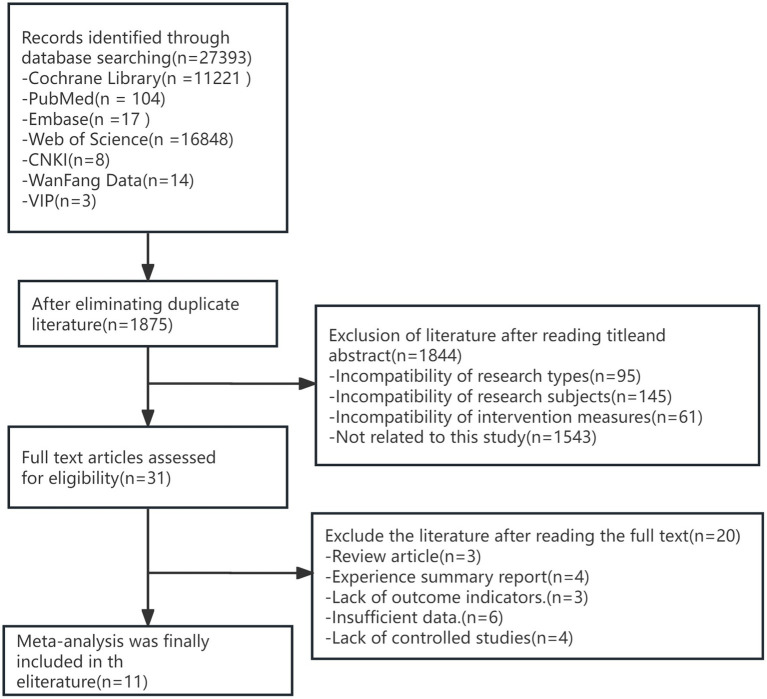
Flowchart of the search strategy.

After screening, our review included 4 RCTs (23–26) and 7 CSs (27–33). Initially, the search identified 27,393 studies. The titles of the initially retrieved studies were imported into NoteExpress, and 1,875 duplicate studies were removed. Then, by screening titles and abstracts, 1844 studies that did not meet the inclusion criteria were excluded, leaving 31 studies. Full-text review of the 31 studies led to the exclusion of 20 studies for the following reasons: review articles (*n* = 3), case reports (*n* = 4), lack of primary outcomes (*n* = 3), insufficient data (*n* = 6), and lack of a control group (*n* = 4) (Figure 1).

### 3.2 Characteristics of included studies

The included studies comprised 1 theoretical course trial (31) and 10 practical course trials (23–30, 32, 33). 9 studies were related to graduates (23–27, 29, 31–33), and 2 studies were related to three-year colleges (28, 30). 3 studies were related to clinical majors (23, 25, 32), and 8 studies were related to nursing majors (24, 26–31, 33). The included studies covered 4 studies on critical care medicine knowledge (23, 25, 26, 32), 3 studies on neurosurgery (24, 29, 30), 2 studies on fundamentals of nursing (31, 33), 1 study on operating room (27), and 1 study on obstetrics (28). The characteristics of the included studies are presented in Table 2.

**Table 2:** Main characteristics of the included studies in the current meta-analysis.

References	Study design	Sample size (cognitive learning theory)	Sample size (LBL)	Population	Majors	Course name	Course type	Outcome measures	RoB2
Yu HT et al. (23)	RCT	35	35	Graduates	Clinical Expertise	Critical Care Medicine	Practice	KES,PS,TS,LC	B
Ye D et al. (24)	RCT	34	34	Graduates	Nursing	Surgery(neurosurgery)	Practice	KES,PS,TS,LC	B
Zhang YJ et al. (25)	RCT	30	30	Graduates	Clinical Expertise	Critical Care Medicine	Practice	KES,PS,TS,LC	B
Li SY et al. (27)	CS	65	61	Graduates	Nursing	Operating room	Practice	KES,PS,LC	B
Yang ZF et al. (28)	CS	54	54	three year college	Nursing	Obstetrics	Practice	KES,TS	B
Wang DM et al. (29)	CS	28	27	Graduates	Nursing	Surgery (neurosurgery)	Practice	KES,PS,TS	B
Wang C et al. (26)	RCT	21	21	Graduates	Nursing	Critical Care Medicine	Practice	KES,PS	B
Wang Y et al. (30)	CS	52	60	three year college	Nursing	Surgery(neurosurgery)	Practice	KES,PS,TS	B
Miner A et al. (31)	CS	37	31	Graduates	Nursing	Fundamentals of Nursing	Theory	KES,TS	B
Bashir K et al. (32)	CS	19	19	Graduates	Clinical Expertise	Critical Care Medicine	Practice	KES,PS	B
Yuliawan D et al. (33)	CS	39	39	Graduates	Nursing	Fundamentals of Nursing	Practice	KES	B

CS, Cohort study; RCT, randomized controlled trial; LBL, lecture-based learning; KES, knowledge examination score; PS, practice score; LC, learning compliance; TS, teaching satisfaction.

RoB 1.0 B indicates a moderate risk of bias in the study.

### 3.3 Risk of bias in included studies

**Figure 2:**
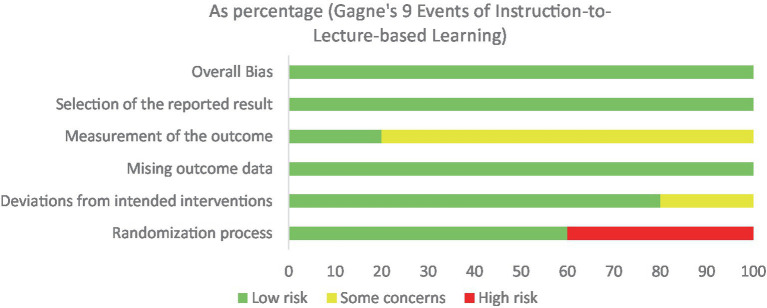
Resultsofriskbiasassessment.

Among the 4 randomized controlled trials (23–26), 1 study (25) had a moderate risk of bias for Deviations from intended interventions and Measurement of the outcome. Still, the overall bias was low. Two studies (23, 26), the outcome measurement was moderate risk, while the overall bias was low. One study (29) had a high-risk Randomization process, but the overall bias was low. Another study (24) had a high-risk Randomization process and a moderate-risk outcome measurement, with the overall bias being low-risk. See Figure 2.

Among the seven non-randomized controlled trials (27–33), two studies (27, 28) had a moderate risk of bias for intervention classification and data missingness, with an overall moderate risk of bias. The remaining four studies had a low overall risk of bias. See Table 3.

**Table 3:** Results of bias risk assessment of non-randomized controlled trials.

References	①	②	③	④	⑤	⑥	⑦	⑧
Bashir K et al. (32)	Low	Low	Low	Low	Low	Low	Low	Low
Li SY et al. (27)	Low	Low	Moderate	Low	Moderate	Low	Low	Moderate
Miner A et al. (31)	Low	Low	Low	Low	Low	Low	Low	Low
Wang Y et al. (30)	Low	Low	Low	Low	Low	Low	Low	Low
Yang ZF et al. (28)	Low	Low	Moderate	Low	Moderate	Low	Low	Moderate
Yuliawan D et al. (33)	Low	Low	Low	Low	Low	Low	Low	Low

① Confounding bias; ② Selection bias of participants; ③ Classification bias of interventions; ④ Deviation bias from intended interventions; ⑤ Bias due to missing data; ⑥ Bias in measurement of outcomes; ⑦ Selective reporting bias; ⑧ Overall bias.

### 3.4 Results of meta-analysis

#### 3.4.1 A meta-analysis of knowledge examination score

**Figure 3:**
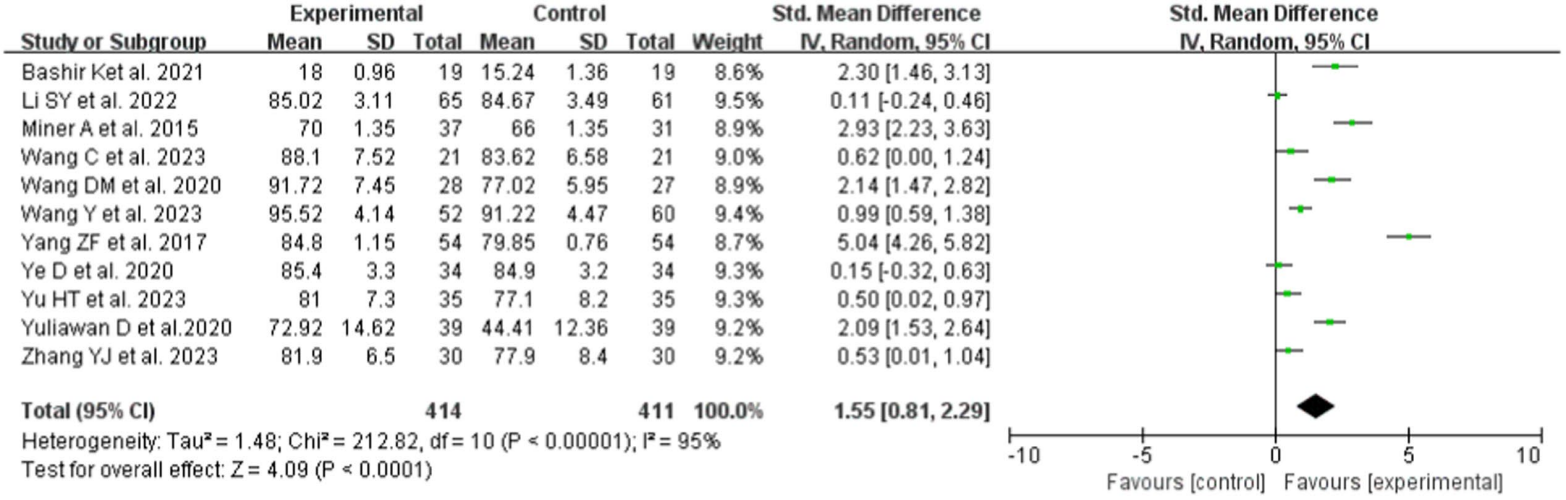
Forest plot of KES for cognitive learning theory compared with LBL.

All studies reported the effectiveness of Gagné’s 9 Events of Instruction on KES. The pooled results (SMD 1.55, 95% CI: 0.81 to 2.29; *p* < 0.00001) showed that the KES of the intervention group was significantly higher than that of the LBL teaching method. Due to the high heterogeneity (*p* < 0.00001, I^2^ = 95% > 50%), a random-effects model was used for the meta-analysis (Figure 3).

#### 3.4.2 A meta-analysis of practice score

**Figure 4:**
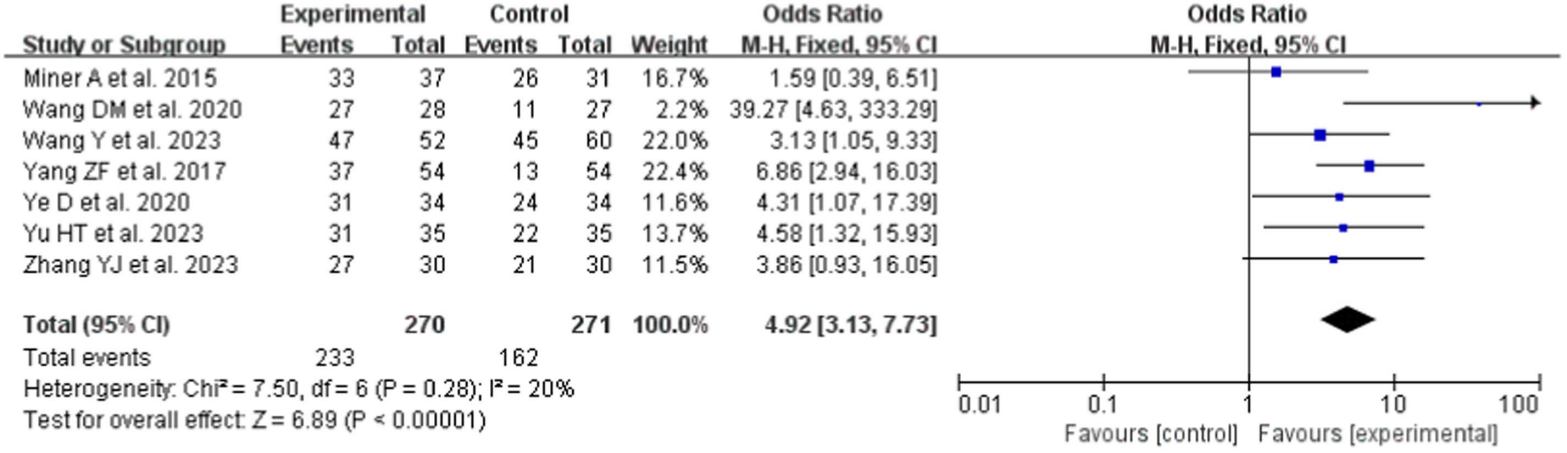
Forest plot of PS for cognitive learning theory compared with LBL.

Among the 11 studies, 8 (23–27, 29, 30, 32) provided PS data before and after the intervention, which was included in the meta-analysis. The pooled effect size of these studies (SMD 1.83, 95% CI: 1.19 to 2.47; *p* < 0.00001) showed that the PS of the intervention group was significantly higher than that of the LBL teaching method. Due to significant statistical heterogeneity between the studies (*p* < 0.00001, I^2^ = 90% > 50%), a random-effects model was used for the meta-analysis (Figure 4).

#### 3.4.3 Meta-analysis of learning compliance

**Figure 5:**
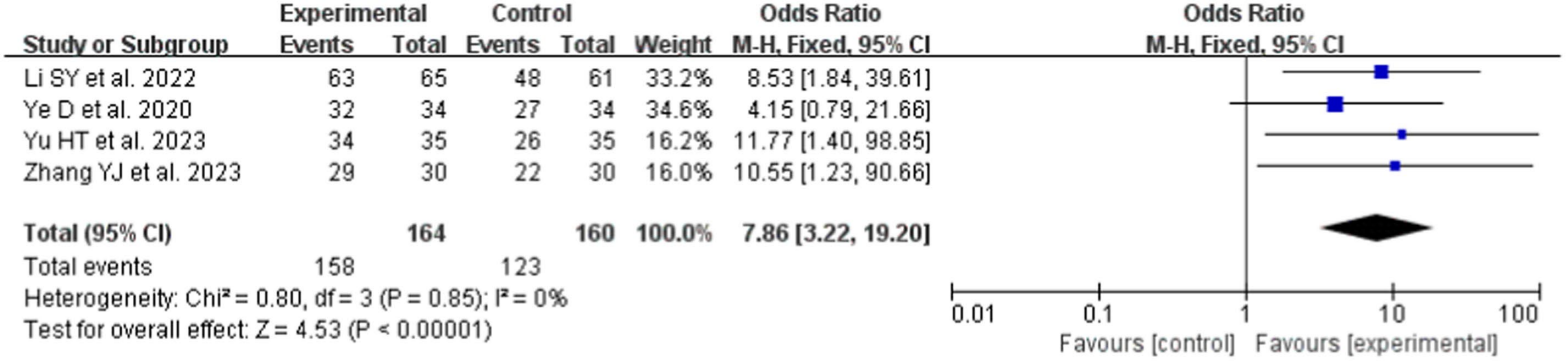
Forest plot of LC for cognitive learning theory compared with LBL.

Four studies (19, 21, 26, 34) reported the effectiveness of Gagné’s 9 Events of Instruction teaching strategy on LC. Compared to LBL, the intervention group showed significantly higher LC (OR = 4.92, 95% CI: 3.13–7.73; *p* < 0.0001). A fixed-effects model was adopted for the meta-analysis (Figure 5) due to the absence of heterogeneity (*p* = 0.28, I^2^ = 20%).

#### 3.4.4 A meta-analysis of teaching satisfaction

**Figure 6:**
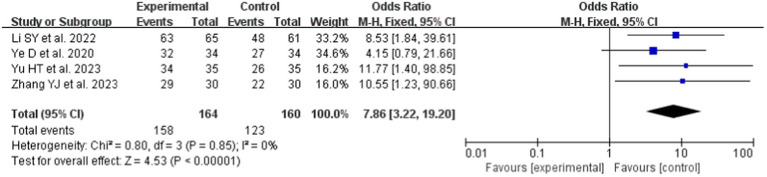
Forest plot of TS for cognitive learning theory compared with LBL.

In this study, 8 studies (19, 21, 22, 27–29, 31, 34) provided data on TS before and after the intervention, which were included in the meta-analysis. Compared to LBL teaching (OR = 7.86, 95% CI: 3.22–19.20; *p* < 0.0001), the LC under the Gagné’s 9 Events of Instruction teaching strategy was significantly higher. A fixed-effects model was employed for the meta-analysis (Figure 6) due to the absence of heterogeneity (*p* = 0.85, I^2^ = 0%).

#### 3.4.5 Subgroup analysis

Given the high heterogeneity observed in KES and PS studies, this may be related to five factors: study design, training levels, course type, course contents, and majors. We conducted subgroup analyses of the relevant literature to investigate whether these factors influenced heterogeneity in KES and PS.

The results showed that in the study design subgroup analysis for KES (Table 4), the heterogeneity in the RCT group was eliminated (I^2^ = 0%), while heterogeneity in the CS group remained substantial (I^2^ = 95%). No significant improvement in heterogeneity was observed in other subgroups. For PS subgroup analyses (Table 5), no subgroup demonstrated significant heterogeneity reduction. The forest plots for subgroup analyses are provided in Supplementary Figure S3.

**Table 4:** Subgroup analyses of KES in this meta-analysis.

Study characteristics	Participants			Test for heterogeneity			Test for effect		Subgroup
	Studies	cognitive learning theory	LBL	I^2^ (%)	Chi^2^ test	*p*-value	(SMD [CI])	*p*-value	Statistics, *p*-value
1. Study design									
Randomized controlled trial (RCT)	4	120	120	0	1.88	=0.60	0.43 [0.17,0.68]	=0.001	9.15, *p* = 0.002
Cohort study (CS)	7	294	291	97	173.48	<0.00001	2.20 [1.08,3.32]	=0.0001	
Total	11	414	411	95	212.82	<0.00001	1.55 [0.81,2.29]	<0.0001	
2. Training levels									
Undergraduates	9	308	297	93	108.61	<0.00001	1.23 [0.56, 1.89]	=0.0003	0.74, *p* = 0.39
Three year college	2	106	114	99	82.32	<0.00001	3.00 [0.96, 6.97]	=0.14	
Total	11	414	411	95	212.82	<0.00001	1.55 [0.81, 2.29]	<0.0001	
3. Course type									
Theory course	1	37	31				2.93 [2.23, 3.63]	<0.00001	8.50, *p* = 0.004
Practice course	10	377	380	95	183.60	<0.00001	1.41 [0.66, 2.16]	=0.0002	
Total	11	414	411	95	212.82	<0.00001	1.55 [0.81, 2.29]	=0.004	
4. Course contents									
Critical Care Medicine	4	105	105	80	15.05	=0.002	0.92 [0.25, 1.58]	=0.007	139.75, *p* < 0.00001
Surgery (neurosurgery)	3	114	121	91	22.83	<0.00001	1.07 [0.09,2.05]	=0.03	
Fundamentals of Nursing	2	76	70	71	3.44	=0.006	2.48 [1.65,3.31]	<0.00001	
Operating Room	1	65	61				0.11 [−0.24,0.46]	=0.55	
Obstetrics	1	54	54				5.04 [4.26, 5.82]	<0.00001	
Total	11	414	411	95	212.82	<0.00001	1.55 [0.81, 2.29]	<0.0001	
5. Majors									
Nursing	8	330	327	96	194.07	<0.00001	1.73 [0.75, 2.71]	=0.0005	1.01, *p* = 0.31
Clinical expertise	3	84	84	87	14.87	=0.0006	1.04 [0.11, 1.96]	=0.03	
Total	11	414	411	95	212.82	<0.00001	1.55 [0.81, 2.29]	=0.31	

**Table 5:** Subgroup analyses of PS in this meta-analysis.

Study characteristics	Participants			Test for heterogeneity			Test for effect		Subgroup
	Studies	cognitive learning theory	LBL	I^2^ (%)	Chi^2^ test	*p*-value	(SMD [CI])	*p*-value	Statistics, *p*-value
1. Study design									
Randomized controlled trial (RCT)	4	120	120	92	36.13	<0.00001	1.39 [0.37,2.41]	=0.008	0.85, *p* = 0.36
Cohort study (CS)	4	164	167	84	18.57	=0.0003	1.97 [1.27,2.66]	<0.00001	
Total	8	284	287	89	61.82	<0.00001	1.68 [1.09,2.27]	<0.00001	
2. Training levels									
Undergraduates	7	232	227	90	57.78	<0.00001	1.78 [1.08,2.48]	<0.00001	2.68, *p* = 0.10
Three year college	1	52	60				1.11 [0.71,1.51]	<0.00001	
Total	8	284	287	89	61.82	<0.00001	1.68 [1.09,2.27]	<0.00001	
3. Course contents									
Critical care medicine	4	105	105	94	48.97	<0.00001	1.80 [0.43,3.16]	=0.01	1.44, *p* = 0.49
Surgery (neurosurgery)	3	114	121	72	7.15	=0.03	1.49 [0.92,2.07]	<0.00001	
Operating room	1	65	61				1.93 [1.50, 2.35]	<0.00001	
Total	8	284	287	89	61.82	<0.00001	1.68 [1.09,2.27]	<0.00001	
4. Majors									
Nursing	5	200	203	75	16.25	=0.03	1.75 [1.26, 2.23]	<0.00001	0.04, P = 0.85
Clinical expertise	3	84	84	95	39.95	<0.00001	1.58 [−0.08, 3.23]	<0.00001	
Total	8	284	287	89	61.82	<0.00001	1.68 [1.09, 2.27]	<0.00001	

#### 3.4.6 Publication bias

**Figure 7:**
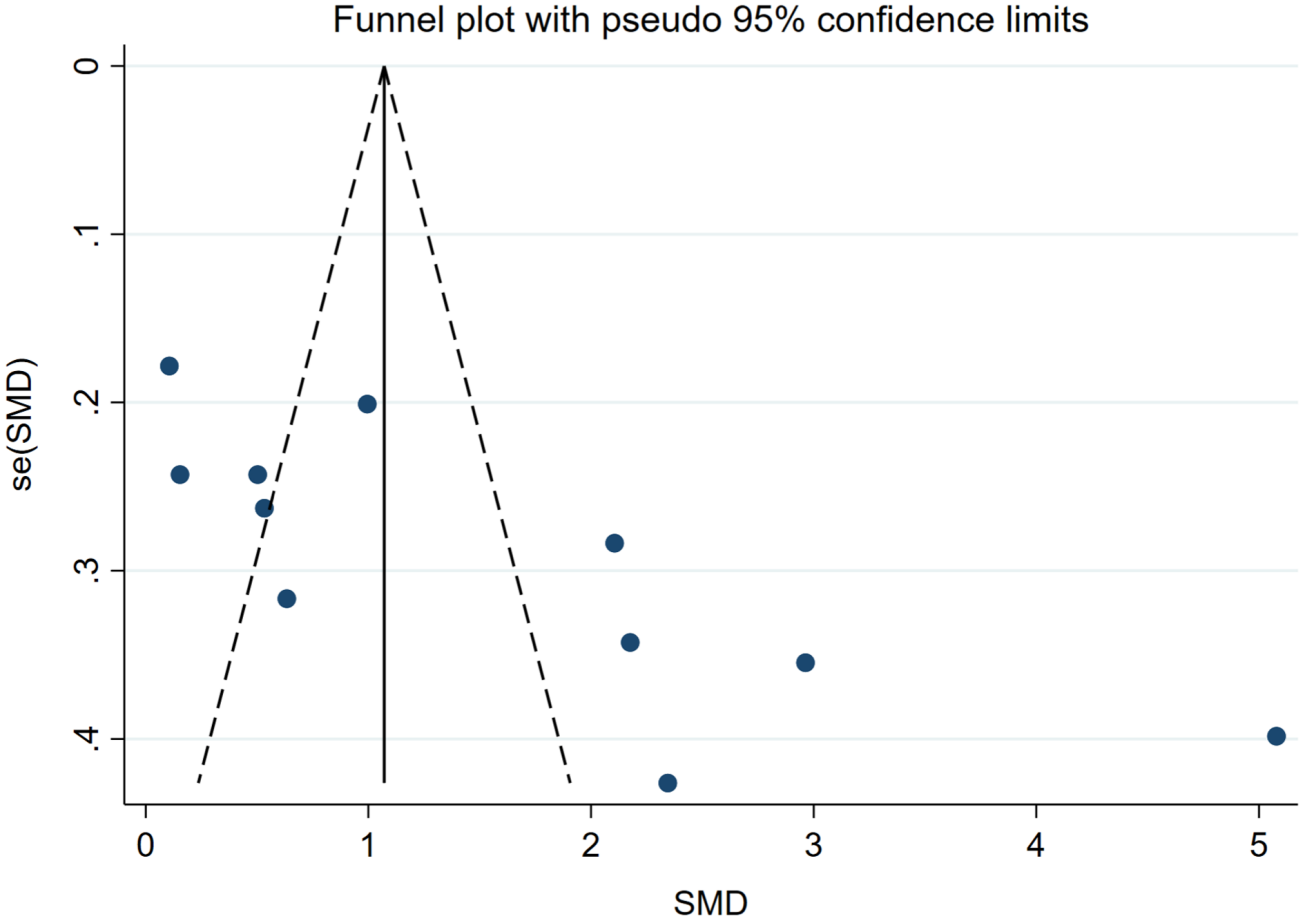
Publication bias of KES.

Due to the inclusion of fewer than 10 studies reporting data on PS, LC, and TS, funnel plots could not be generated to assess publication bias for these outcomes. Therefore, publication bias analysis was only performed for KES using a funnel plot. The study revealed that the funnel plot exhibited near-symmetry, indicating negligible evidence of significant publication bias (Figure 7).

#### 3.4.7 Sensitivity analysis

**Figure 8:**
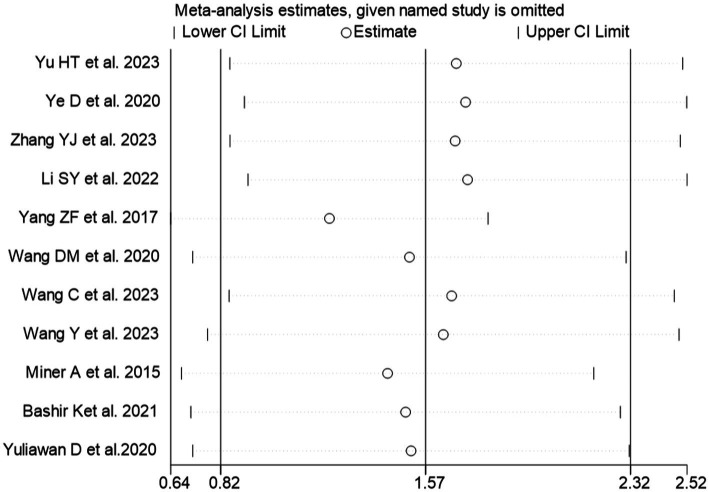
Sensitive analysis of KES.

A sensitivity analysis was performed for KES using the leave-one-out method (Figure 8). Upon exclusion of any individual study, the direction and magnitude of the pooled estimates remained consistent without significant changes, indicating the robustness of the meta-analysis results.

## 4 Discussion

Contemporary health professions education faces numerous challenges, requiring learners not only to master clinical expertise and skills but also to develop capabilities to adapt to medical advancements and innovations (35). Traditional health professions education predominantly adopts an “educator-led, learner-passive” model (36), which neglects learners’ central role and self-directed learning abilities, often resulting in learners’ failure to apply theoretical knowledge in clinical practice and a significant disconnect between theory and application. Although Gagné’s 9 Events of Instruction has demonstrated positive educational outcomes in higher education systems (37), its implementation in health professions education remains nascent. Primarily, health professions education emphasizes the transmission of foundational knowledge and technical skills while insufficiently fostering learners’ autonomous learning and innovative capacities, leading educators to prefer conventional lecturing methods when selecting pedagogical strategies. Secondly, open and interactive teaching approaches demand educators’ professional competence and advanced classroom management skills, requiring substantial teaching resources to guide and regulate learners’ progress in open-learning environments effectively. Finally, significant disparities exist in medical curricula standards and educational levels across different regions. Consequently, enhancing the quality of medical talent cultivation and improving the effectiveness of health professions education constitutes a critical challenge in contemporary health professions education.

The meta-analysis results demonstrated that compared to the LBL group, Gagné’s 9 Events of Instruction group exhibited significantly higher scores in KES, PS, LC, and TS, indicating that this instructional approach enables learners to transform acquired theoretical knowledge and practical skills into procedural knowledge of intellectual skills and cognitive strategies applicable in clinical practice. Furthermore, by systematically dividing the learning process into nine stages—Gain Attention, Inform Learners of Objectives, Stimulate Recall of Prior Learning, Present Stimulus, Provide Learner Guidance, Elicit Performance, Provide Feedback, Assess Performance, and Enhance Retention and Transfer—the Gagné framework achieves a “spiral learning progression” through iterative guidance, evaluation, and re-evaluation, thereby assisting educators in innovating pedagogical concepts and dynamically adjusting instructional pacing (38). Additionally, given the extensive knowledge domains in health professions education, the LBL method fails to encourage learners to explore their attention, learning, memory, and cognition processes. The meta-analysis revealed that learners in Gagné’s 9 Events of Instruction group demonstrated superior knowledge mastery and enhanced self-directed learning capabilities compared to their LBL counterparts.

The combined effect sizes of KES (SMD 1.55, 95% CI: 0.81–2.29; *p* < 0.00001), PS (SMD 1.83, 95% CI: 1.19–2.47; *p* < 0.00001), LC (OR 4.92, 95% CI: 3.13–7.73; *p* < 0.0001), and TS (OR 7.86, 95% CI: 3.22–19.20; *p* < 0.0001) demonstrate that Gagné’s 9 Events of Instruction is significantly more effective than traditional LBL pedagogy. This effectiveness likely stems from Gagné’s framework, stimulating learning through external cues, fostering learners’ anticipation for new knowledge and skills, and generating intrinsic motivation (39). The approach proactively mobilizes learners’ self-directed learning initiatives and subjective agency, enabling them to derive intellectual enjoyment from theoretical studies and enhance educational outcomes. Additionally, within conventional teaching environments, learners’ curiosity about this novel instructional model intensifies their learning motivation and engagement, contributing to superior KES and TS achievements. By shifting the paradigm from educator-centered “teaching” to learner-centered “learning,” Gagné’s framework positions learners as active constructors of knowledge and meaning (40). Educators in this model transition from passive knowledge dissemination to prioritizing the stimulation of learning interests, encouraging learners to explore their cognitive processes of attention, knowledge acquisition, memory retention, and critical thinking. Consequently, the intervention group’s pedagogical superiority over traditional LBL methods becomes markedly evident.

When analyzing the KES and PS outcome measures, significant heterogeneity was observed among the included studies. To explore this phenomenon, the outcome measures of KES and PS were stratified into five subgroups: Study design, Training levels, Course type, Course contents, and Majors. Subgroup analysis of the Study design for KES revealed no heterogeneity in the RCT group (I^2^ = 0%), suggesting that heterogeneity in KES may be associated with the Study design. However, significant heterogeneity persisted in KES across subgroups of Study design, Training levels, Course type, and Majors, indicating the minimal influence of these factors on KES heterogeneity.

For PS, subgroup analyses of Training levels, Study design, Course type, Course contents, and Majors also demonstrated persistent heterogeneity, highlighting complex variability across these dimensions. Consequently, future studies should investigate additional potential sources of heterogeneity. Significant heterogeneity in KES and PS remained unresolved after subgroup analyses. Possible explanations include variations in educational proficiency, curriculum structure, assessment difficulty, and learners’ academic capabilities. The intricate interactions among these factors may contribute to outcome variability, underscoring the necessity to rigorously account for these influences in future research designs to enhance consistency and reliability.

In recent years, Gagné’s 9 Events of Instruction has been extensively trialed in higher education and demonstrated efficacy in enhancing health professions education outcomes (41). This instructional strategy employs a nine-phase framework to design learning activities, which helps educators deconstruct and analyze instructional processes to improve learners’ academic achievements in health professions education and supports the establishment of innovative curriculum development concepts. It promotes course innovation, achieves outcome-oriented curriculum construction goals, and fosters the formation of multi-format, diversified instructional models.

However, this systematic review and meta-analysis have several limitations: ① The included studies generally exhibited relatively low quality and small sample sizes, necessitating the inclusion and publication of more large-scale, multi-center, high-quality research. ② Significant heterogeneity in KES and PS may be attributed to factors such as variations in course types, educator proficiency, instructional design, assessment content, and learners’ academic capabilities. ③ Methodological constraints inherent to educational interventions—specifically, the inability to implement allocation concealment or blinding for participants and instructors—may have introduced selection and information biases. ④ Heterogeneity in outcome evaluation metrics across studies limited the analysis of additional shared indicators. ⑤ While standardization challenges across rating systems were mitigated through SMD calculations, incomplete reporting of standard deviations or scoring details in some studies may compromise effect size precision. Future research should adopt standardized rating systems and publicly share raw data to facilitate robust cross-study comparisons.

## 5 Conclusion

This systematic review and meta-analysis demonstrates that, compared to LBL, Gagne’s 9 Events of Instruction exhibits significant advantages in health professions education. The method effectively enhances learners’ KES, PS, LC, and TS, with these benefits likely stemming from its systematic design that strengthens cognitive processes and stimulates learning motivation. Despite existing study heterogeneity (e.g., variations in course types and evaluation criteria) and limitations in research quality (such as small sample sizes and methodological biases), the results still support the application value of Gagne’s 9 Events of Instruction in health professions education. Future research should prioritize high-quality, multicenter randomized controlled trials to further validate its long-term efficacy and optimize instructional design, addressing challenges in integrating theory and practice within medical education.

## Data Availability

The original contributions presented in the study are included in the article/Supplementary material, further inquiries can be directed to the corresponding author.
