# Supplementary material for: Effectiveness of Gagné’s 9 Events of Instruction in health professions education: a systematic review and meta-analysis
Source: Front Med (Lausanne). 2025 Apr 8;12:1522830. doi: 10.3389/fmed.2025.1522830 (PMC12011725; doi:10.3389/fmed.2025.1522830)
Supplement: Supplementary file 1 [file Table_1.docx]

| **Section and Topic** | **Item #** | **Checklist item** | **Location where item is reported** |
| --- | --- | --- | --- |
| **TITLE** | | |  |
| Title | 1 | The report was identified as a meta-analysis and systematic review | 1 |
| **ABSTRACT** | | |  |
| Abstract | 2 | Structured abstracts include Objective, methods, results, and conclusions. | 1 |
| **INTRODUCTION** | | |  |
| Rationale | 3 | The description in the introduction | 2-3 |
| Objectives | 4 | Explained in the introduction | 2-3 |
| **METHODS** | | |  |
| Eligibility criteria | 5 | The specified research features ( PICOS ) and report features ( such as retrieval time, language and publication status ) were used as the criteria for inclusion in the study, and explanations were given. | 4 |
| Information sources | 6 | Describe the source of all the literature information for each search and the final search results. | 5 |
| Search strategy | 7 | Develop search strategies based on PICOS criteria ( population, intervention, control, results, and study design ). | 3 |
| Selection process | 8 | Two researchers identified and selected data in detail and independently from the study, and these differences were resolved by consensus or collaboration with a third member of the research team. | 4 |
| Data collection process | 9 | Two researchers extracted data from the study in detail, and the differences were resolved by consensus or in collaboration with a third member of the research team. | 4 |
| Data items | 10a | The surname, year, Study design, sample size, education level, majors, course name, course type and outcome measures of the first author to be collected are described in detail. The primary outcome measures were knowledge examination score ( KES ), practice score ( PS ), secondary outcome measures were learning compliance ( LC ) and teaching satisfaction ( TS ). | 4 |
|  | 10b | participant :Medical students | 4 |
| Study risk of bias assessment | 11 | Independently assess the quality of included studies using the Cochrane risk of bias tool. Funnel plot was used to independently evaluate whether there was publication bias in the results of this study. The symmetrical distribution of each index in the funnel plot indicates that there is no publication bias. | 4 |
| Effect measures | 12 | For continuous data : KES,PS, we use Standardized Mean Difference (SMD) and related 95 % confidence interval ( CI ) to estimate the effect size. For the categorical variable : LC,TS, we used Odds Ratio (OR) and 95 % confidence interval ( CI ) for statistical evaluation. | 4 |
| Synthesis methods | 13a | Describe the processes used to decide which studies were eligible for each synthesis (e.g. tabulating the study intervention characteristics and comparing against the planned groups for each synthesis (item #5)). | 6-7 |
|  | 13b | Describe any methods required to prepare the data for presentation or synthesis, such as handling of missing summary statistics, or data conversions. |  |
|  | 13c | Use Excel for data statistics | 4 |
|  | 13d | Using RevMan 5.4 and Stata 17.0 software. KES and PS were considered continuous variables, and the effect size was estimated using mean difference (MD) and corresponding 95% confidence interval (CI). LC and TS were classified variables, and statistical evaluation was performed using relative risk (OR) and corresponding 95% CI. A significance level of P <0.05 was considered statistically significant . Heterogeneity testing was performed for the included studies. If no significant heterogeneity was observed (I^2^ < 50%, P ≥ 0.05), a fixed-effect model was used for the meta-analysis. If heterogeneity was present (I^2^ ≥ 50%, P < 0.05), a random-effects model was used | 4-5 |
|  | 13e | Subgroup analysis of KES and PS was performed. | 10-13 |
|  | 13f | If I^2^ < 50%, a fixed-effects model was used for analysis, indicating low to moderate heterogeneity or no statistical heterogeneity in the studies. If I^2^ ≥ 50%, a random-effects model was used for analysis. | 5 |
| Reporting bias assessment | 14 | Two researchers independently assessed the quality of the included studies using the Cochrane Risk of Bias Tool. | 4 |
| Certainty assessment | 15 | Describes the methods used to evaluate the quality of evidence for each outcome. |  |
| **RESULTS** | | |  |
| Study selection | 16a | Flow chart : retrieval and screening process, the number of records retrieved to the number of studies finally included. | 5 |
|  | 16b | Explain those studies that met the inclusion criteria but were excluded. | 5 |
| Study characteristics | 17 | Table 2 shows the characteristics of each study extracted. | 6-7 |
| Risk of bias in studies | 18 | Risk of bias results of included studies | 7-8 |
| Results of individual studies | 19 | Meta-analysis of outcome indicators : KES ( Fig.4 ), PS ( Fig.5 ), LC ( Fig.6 ), TS ( Fig.7 ). | 9-10 |
| Results of syntheses | 20a | The symmetry of KES funnel plot is good.The quality of the included studies was evaluated. | 8 |
|  | 20b | knowledge examination score (KES) (SMD 1.55, 95% CI: 0.81 to 2.29; P < 0.00001), practice score (PS) (SMD 1.83, 95% CI: 1.19 to 2.47; P < 0.00001), learning compliance (LC) (OR 4.92, 95% CI: 3.13 to 7.73; P < 0.0001), and teaching satisfaction (TS) (OR 7.86, 95% CI: 3.22 to 19.20; P < 0.0001). | 9-10 |
|  | 20c | Subgroup analysis of KES and PS was performed. | 10 |
|  | 20d | Sensitivity analysis was conducted on the knowledge examination scores (KES) using the one-at-a-time exclusion method | 13 |
| Reporting biases | 21 | Risk of bias in the included studies | 8 |
| Certainty of evidence | 22 | The results of confidence evaluation are given. | 9-10 |
| **DISCUSSION** | | |  |
| Discussion | 23a | Provide a general interpretation of the results in the context of other evidence. | 14-17 |
|  | 23b | Discuss any limitations of the evidence included in the review. | 16-17 |
|  | 23c | Discuss any limitations of the review processes used. | 16-17 |
|  | 23d | Discuss implications of the results for practice, policy, and future research. | 17 |
| **OTHER INFORMATION** | | |  |
| Registration and protocol | 24a | Not applicable. | 3 |
|  | 24b | No agreement prepared | 3 |
|  | 24c | No agreement prepared | 3 |
| Support | 25 | The authors declare that they have no source of funding for the research. | 20 |
| Competing interests | 26 | The authors declare that they have no competing interests. | 20 |
| Availability of data, code and other materials | 27 | The data included in the study and the data used for analysis are provided in the annex. | 20 |

*From:*  Page MJ, McKenzie JE, Bossuyt PM, Boutron I, Hoffmann TC, Mulrow CD, et al. The PRISMA 2020 statement: an updated guideline for reporting systematic reviews. BMJ 2021;372:n71. doi: 10.1136/bmj.n71

For more information, visit: <http://www.prisma-statement.org/>
