# Supplementary material for: Effectiveness of Gagné’s 9 Events of Instruction in health professions education: a systematic review and meta-analysis
Source: Front Med (Lausanne). 2025 Apr 8;12:1522830. doi: 10.3389/fmed.2025.1522830 (PMC12011725; doi:10.3389/fmed.2025.1522830)
Supplement: Supplementary file 2 [file Table_2.docx]

**S 2 Search strategys**

| Database | Search Details | Results |
| --- | --- | --- |
| PubMed | (((((Gagne's 9 Events of Instruction[Title/Abstract]) OR (Gagne’s Nine Steps of Instructional Design[Title/Abstract])) OR (Gagne’s Model of Instructional Design[Title/Abstract])) OR (Gagne’s Model of Instructional Design[Title/Abstract])) OR (nine events[Title/Abstract])) AND (((((Education) OR (Nursing)) OR (clinical)) OR (medicine)) OR (Student)) | 104 |
| web of science | #1((((TS=(Gagne's 9 Events of Instruction)) OR TS=(Gagne’s Nine Steps of Instructional Design)) OR TS=(Gagne’s Model of Instructional Design)) OR TS=(nine events))  #2((((AB=(Education)) OR AB=(Nursing)) OR AB=(clinical)) OR AB=(medicine)) OR AB=(Student) and Preprint Citation Index (Exclude – Database)  #1 AND #2 | 16848 |
| Embase | #14 #6 AND #13 17  #13 #7 AND #8 AND #9 AND #10 AND #11 16343  #11 nursing 3882257  #10 student 627690  #9 medicine 15055007  #8 clinical 17074810  #7 education 2529266  #6 #1 OR #2 OR #3 OR #4 37855  #5 'three-dimensional printing in the preoperative planning of thoracoscopic pulmonary segmentectomy':ti 1  #4 gagne AND model AND of AND instructional AND design 25  #3 gagne AND nine AND steps AND of AND instructional AND design 1  #2 gagne AND 9 AND events AND of AND instruction 6  #1 nine AND events 37834 | 17 |
| cochrane | #1 nine events 11221  #2 Gagne’s Model of Instructional Design 1  #3 Gagne's 9 Events of Instruction 1  #4 Gagne’s Nine Steps of Instructional Design 0  #5 #1 or #2 or #3 or #4 11222  #6 Education 117063  #7 Nursing 55163  #8 clinical 1377232  #9 medicine 359353  #10 Student 32166  #11 #6 or #7 or #8 or #9 or #10 1547860  #12 #5 and #11 10399 | 10399 |
| CNKI | “(主题:加涅教学理论)OR(主题:加涅理论)OR(主题:九段教学法)OR(主题:加涅教学模式)”AND“(篇关摘:医学(模糊))OR(篇关摘:临床医学(模糊))OR(篇关摘:护理学(模糊))OR(篇关摘:专科医学生(模糊))OR(篇关摘:实习生(模糊))” | 8 |
| WanFang Data, | (主题:(加涅教学理论) or 主题:(加涅理论) or 主题:(九段教学法) or 主题:(加涅教学模式)） and (题名或关键词:(医学) or 题名或关键词:(临床医学) or 题名或关键词:(护理学) or 题名或关键词:(专科医学生) or 题名或关键词:(专科医学生)) | 14 |
| VIP databases | ((((题名或关键词=加涅教学理论 OR 题名或关键词=加涅理论) OR 题名或关键词=九段教学法) OR 题名或关键词=加涅教学模式) AND ((((题名或关键词=医学 OR 题名或关键词=临床医学) OR 题名或关键词=护理学) OR 题名或关键词=专科医学生) OR 题名或关键词=实习生)) | 3 |
