# Supplementary material for: Effectiveness of Gagné’s 9 Events of Instruction in health professions education: a systematic review and meta-analysis
Source: Front Med (Lausanne). 2025 Apr 8;12:1522830. doi: 10.3389/fmed.2025.1522830 (PMC12011725; doi:10.3389/fmed.2025.1522830)

**S 3 Subgroup analysis**

**1.Forest plot of subgroup analyses of final knowledge examination score**

1.1 Subgroup analyses of study design


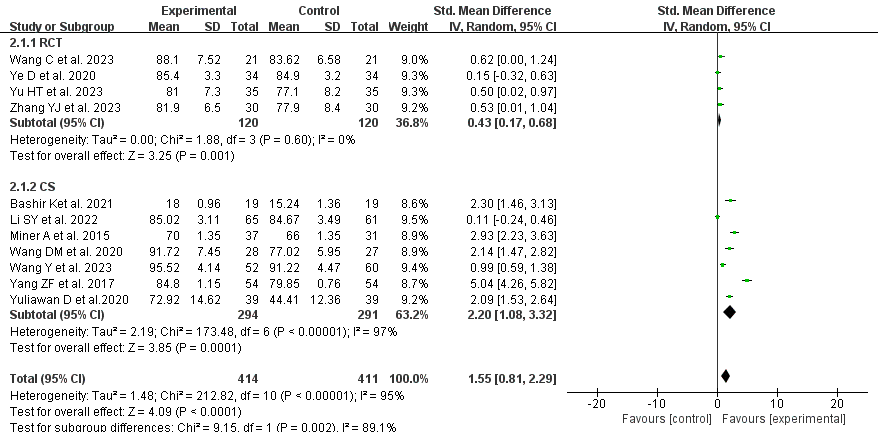


**1.Forest plot of subgroup analyses of final knowledge examination score**

1.2 Subgroup analyses of training levels


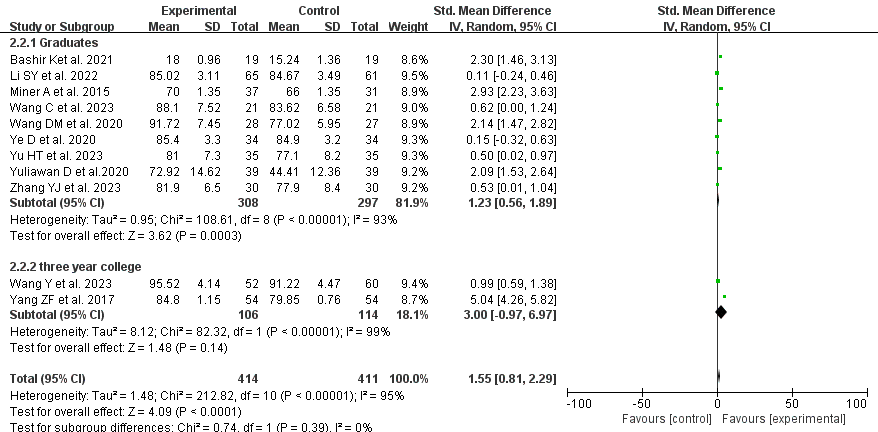


**1. Forest plot of subgroup analyses of final knowledge examination score**

1.3 Subgroup analyses of course type


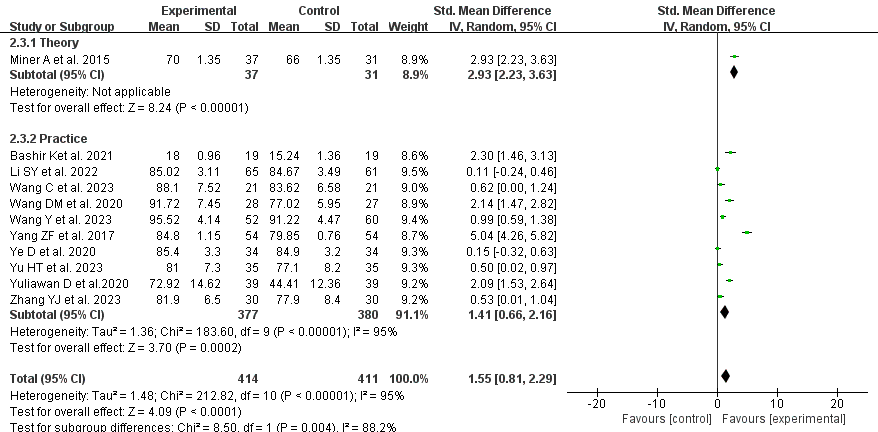


**1.Forest plot of subgroup analyses of final knowledge examination score**

1.4 Subgroup analyses of course contents


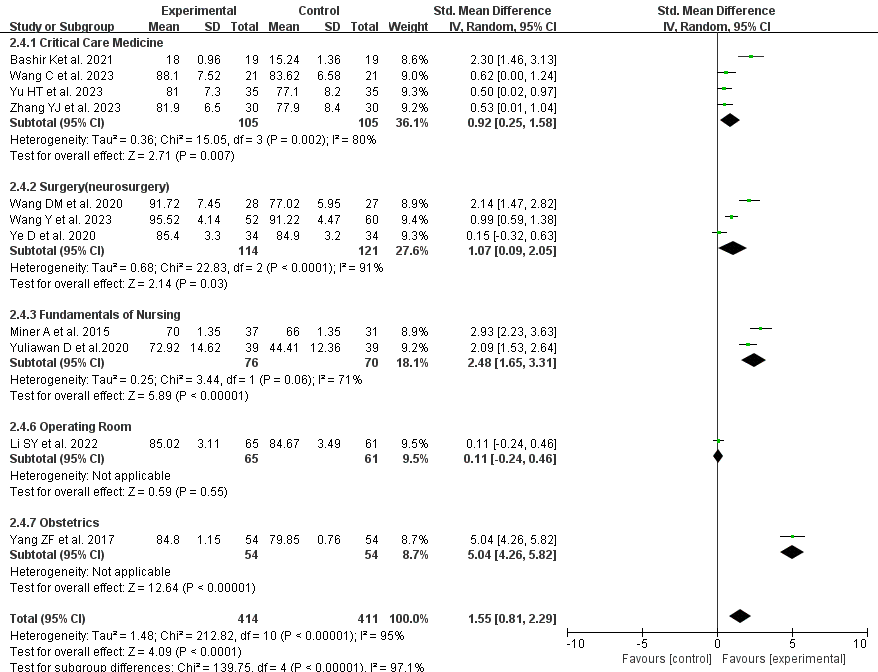


**1.Forest plot of subgroup analyses of final knowledge examination score**

1.5 Subgroup analyses of majors


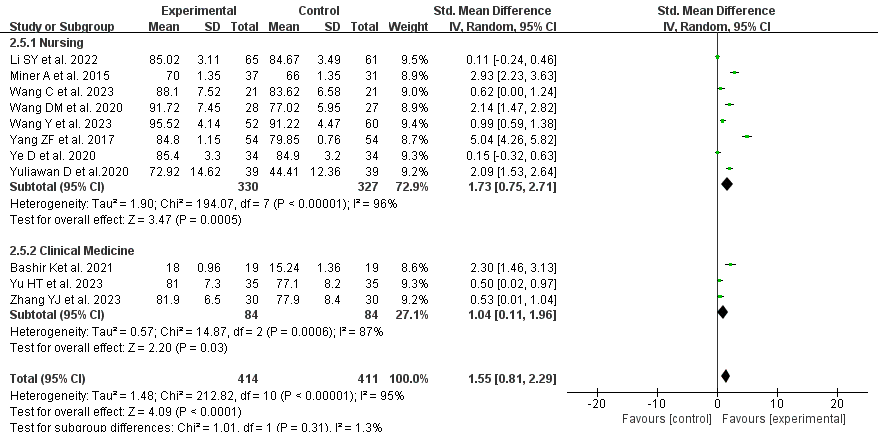


**2.Forest plot of subgroup analyses of practice score**

2.1 Subgroup analyses of study design


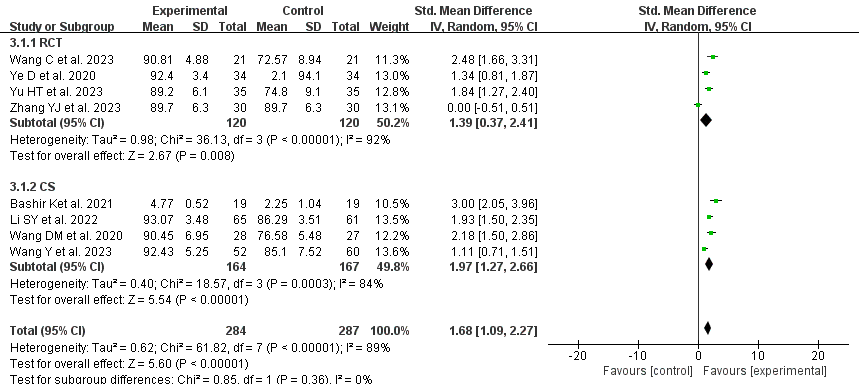


**2.Forest plot of subgroup analyses of practice score**

2.2 Subgroup analyses of training levels


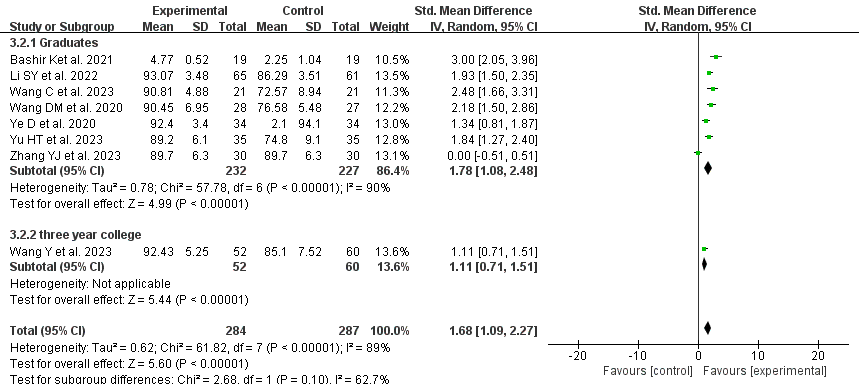


**2.Forest plot of subgroup analyses of practice score**

2.4 Subgroup analyses of course contents


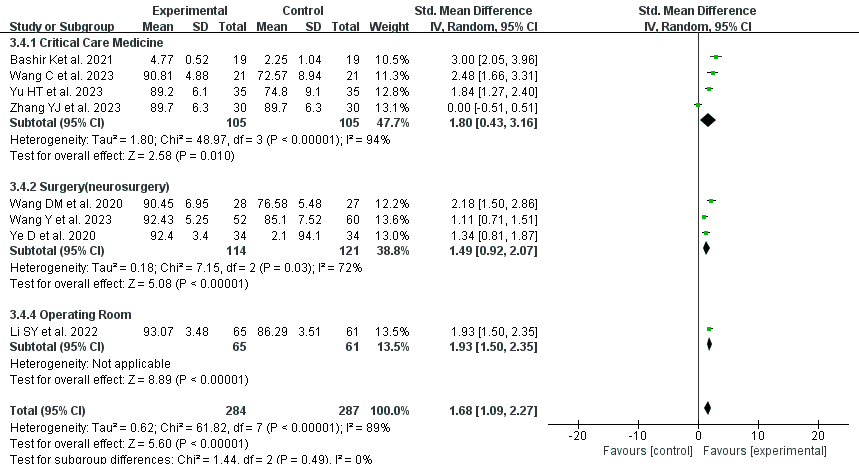


**2.Forest plot of subgroup analyses of practice score**

2.5 Subgroup analyses of majors


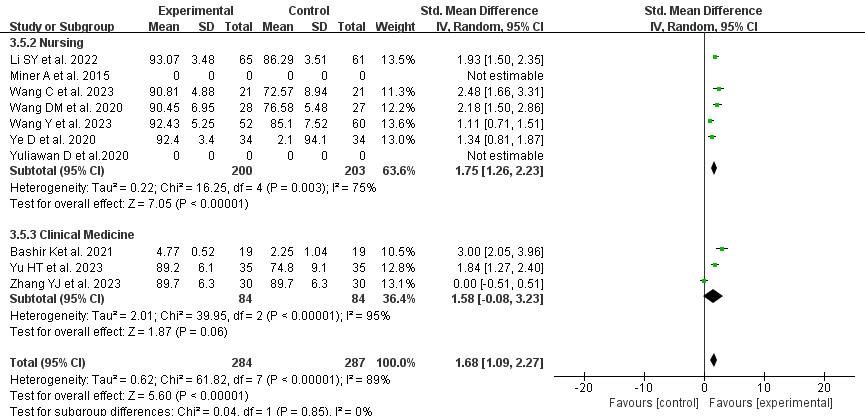

Supplement: Supplementary file 3 [file Data_Sheet_3.docx]
